# A novel TRP channel-related prognostic model of glioma based on transcriptomics and single cell sequencing analysis

**DOI:** 10.1007/s12672-025-04220-5

**Published:** 2025-12-02

**Authors:** Xiaochen Niu, Aijie Guo, Xuanchen Liu, Hao Li, Hongming Ji, Chunhong Wang

**Affiliations:** 1 Department of Neurosurgery, Shanxi Provincial People’s Hospital, Taiyuan, 030012 China; 2 The Fifth Clinical Medical College of Shanxi Medical University, Taiyuan, 030012 China; 3 Shanxi Provincial Key Laboratory of intelligent Brain Tumor, Taiyuan, 030012 China; 4 Shanxi Provincial Key Laboratory of intelligent, big data and digital neurosurgery, Taiyuan, 030012 China; 5 Department of Epidemiology and Biostatistics, West China School of Public Health, West China Fourth Hospital, Sichuan University, Chengdu, 610041 China; 6 Department of Nutrition and Food Hygiene, West China School of Public Health, West China Fourth Hospital, Sichuan University, Chengdu, 610041 China

**Keywords:** Transient receptor potential channels, Glioma, Bioinformatics, Prognosis, Tumor microenvironment

## Abstract

**Background:**

Glioma is the most malignant intracranial tumor. Transient receptor potential (TRP) channel family has been found to be involved in malignant progression of many tumors. However, the relationship between TRP channel-related genes (TCRGs) and glioma remains unclear.

**Methods:**

Gene expression profiles and clinical data of 1,475 glioma patients were obtained from TCGA, CGGA, and GEO databases. Prognostic TCRGs were screened and used to classify the patients. Lasso Cox regression analysis was used to construct a risk model, which was validated in external cohorts, and the patients were stratified into high- and low-risk groups. Immune infiltration and functional enrichment analyses were performed to explore the tumor microenvironment in two groups, while drug sensitivity predictions were conducted. Single-cell RNA sequencing data were analyzed to examine the cell type-specific expression of key model genes. Finally, RT-qPCR was performed on paired glioma and adjacent normal tissues to validate the expression of all model genes.

**Results:**

Thirty-seven differentially expressed TCRGs were identified in glioma, of which 30 were associated with patient survival. Consensus clustering revealed three molecular subtypes with distinct prognoses, immune infiltration, and pathway enrichment. A 10-gene (TRPM6, PRKCB, CAMK2G, ADCY5, HTR2A, P2RY2, MAPK13, BDKRB1, PLA2G4D, and TRPV3) prognostic model stratified patients into high- and low-risk groups with significantly different overall survival, validated in external cohorts. High-risk patients exhibited higher immune cell infiltration and were predicted to be more sensitive to drugs including 5-Fluorouracil, Dasatinib, Gemcitabine, and Rapamycin, whereas low-risk patients were more sensitive to Vorinostat, Lapatinib, Gefitinib, and Osimertinib. Single-cell RNA sequencing showed that TRPV3 was expressed in exhausted CD8^+^ T cells, supporting the model’s relevance to tumor immunity and patient prognosis. RT-qPCR verification indicated that all 10 genes in the model were expressed at lower levels in glioma tissues.

**Conclusion:**

Based on the expression of TCRGs, we conducted the new subtype classification and a prognostic model for glioma, and is expected to provide theoretical basis for the development of new targets.

**Supplementary Information:**

The online version contains supplementary material available at 10.1007/s12672-025-04220-5.

## Introduction

Glioma is highly malignant primary brain tumor originating from glial cells, with an incidence of approximately 5.26 cases per 100,000 people and approximately 17,000 new cases worldwide each year [1]. Glioma is classified into LGG (low grade glioma, WHO Grade 2–3) and GBM (glioblastoma, WHO Grade 4), among which GBM has an extremely poor prognosis and a high mortality rate [2]. At present, the main treatment methods for glioma are safe surgical resection within the largest range, supplemented by radiotherapy and chemotherapy, tumor treating fields therapy, and immune-targeted therapy. These therapies can prolong the median overall survival (OS) of LGG patients to approximately 5–10 years, and the median survival time of GBM can reach approximately 1–2 years, but the overall treatment effect is still unsatisfactory [3]. The main reason is that glioma is very aggressive and heterogeneous, prone to recurrence and progression, and the long-term therapeutic efficacy remains unsatisfactory. With the emergence of precise solutions such as the tumor immune microenvironment, immune checkpoints, tumor-associated macrophages, oncolytic viruses, and gene-targeted therapy, the exploration of more efficient glioma biomarkers has become a new research direction, which is expected to achieve more precise treatment of glioma [4].

Transient receptor potential (TRP) is a multifunctional signaling molecule located on the cell membrane surface that interacts with a variety of ion channels to regulate intracellular and intercellular communication [5]. The TRP channel superfamily consists of six transmembrane cation permeation channels, which function as molecular sensors for various physical and chemical stimuli and are divided into seven subfamilies with different functions according to different homology of amino acid sequence: TRP-A, C, M, ML, N, P and V [6]. The TRP channel family participates in the angiogenesis, invasion and migration of cancer by regulating the proliferation, differentiation and apoptosis of tumor cells and plays a key role in the drug sensitivity of tumor cells [7]. TRP channel-related genes (TCRGs) are abnormally expressed in a variety of tumors [8]. For example, studies have found that TCRGs have a clear role in the proliferation and occurrence of breast cancer cells. The interaction between TRPA1 and TRPV1 promotes the resistance of breast cancer cells to oxidative stress and increases the resistance of tumor cells to chemotherapy drugs. TRPC1 and TRPV4 regulate endothelial cell calcium homeostasis, TRPM is associated with cell invasion, and TRPM4 plays an important role in lymph node spread of well-differentiated tumor cells [9]. Stock K et al. found that the TRPV1 signaling pathway mediates the occurrence of glioma by regulating the calcium concentration of astrocytes in the brain [10].

**Fig. 1:**
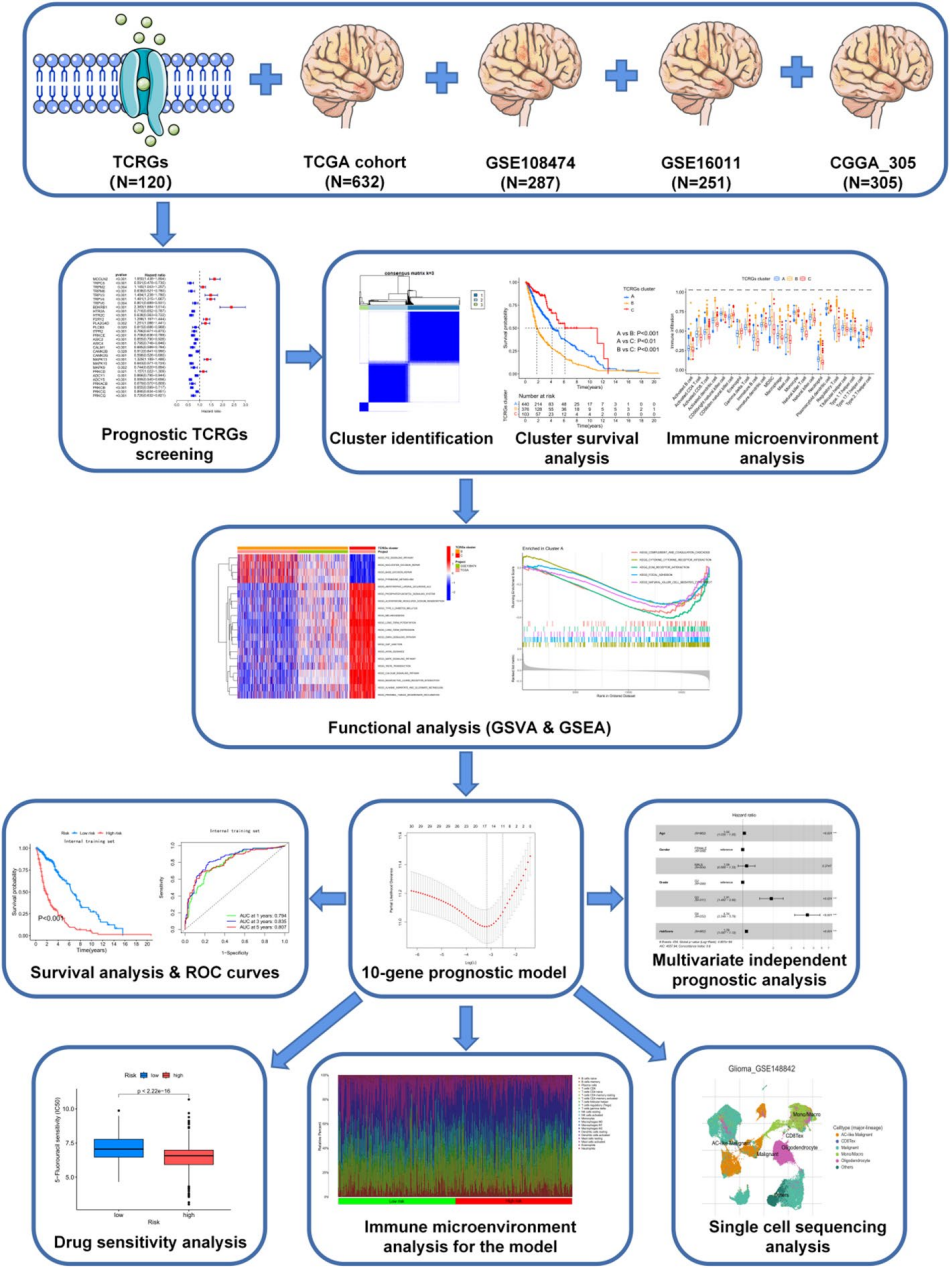
The flow-chart of this study

Based on the above evidence, this study aimed to identify prognostic TCRGs and construct a robust risk model to facilitate glioma stratification, prognosis prediction, and the discovery of potential therapeutic targets (Fig. 1).

## Methods and materials

### Data acquisition

We downloaded tumor tissue sequencing data and clinical information of glioma patients (WHO Grade 2–4) from The Cancer Genome Atlas [11] (TCGA, https://portal.gdc.cancer.gov/), Gene Expression Omnibus [12] (GEO, https://www.ncbi.nlm.nih.gov/geo/) and Chinese Glioma Genome Atlas [13] (CGGA, http://www.cgga.org.cn/). To avoid statistical bias, samples with missing clinical data and OS less than 30 days were excluded, and 1,475 patients were included. The “sva” package [14] in R 4.5.0 (https://www.r-project.org/) was used to eliminate batch effects between the TCGA (*n* = 632) and GSE108474 cohort (*n* = 287) [15]. The gene expression matrices from two cohorts were merged by retaining the intersecting genes across datasets. To minimize non-biological variation, we applied the ComBat function [16] with the dataset source as the batch variable. Before batch correction, quantile normalization was performed on non-TCGA datasets and log2-transformation was applied where appropriate. To evaluate the effectiveness of batch correction, we conducted principal component analysis (PCA) on the combined dataset before and after applying ComBat. Prior to correction, samples clustered predominantly by dataset (i.e., TCGA vs. GSE108474), reflecting strong batch effects. After correction, samples no longer segregated by batch, indicating successful elimination of batch-related variation (Supplementary Fig. 1). The corrected expression matrix was used for all downstream analyses. The GSE16011 (*n* = 251) [17] and CGGA_325 cohorts (*n* = 305) were used as external validation sets to test the model. From the REACTOME_TRP_CHANNELS pathway in the Molecular Signatures Database [18] (MsigDB, http://www.gsea-msigdb.org/gsea/msigdb) and inflammatory mediator regulation of TRP channels in Kyoto Encyclopedia of Genes and Genomes [19] (KEGG, https://www.kegg.jp/), TCRGs were extracted and duplicated genes were deleted. A total of 120 genes were obtained for bioinformatics analysis (Supplementary Table 1).

### Identification of prognostic TCRGs

Using five normal brain tissues of the TCGA cohort and the “limma” package [20] to pair 120 TCRGs. The expression difference between normal brain tissues and tumor was analyzed, and the false discovery rate (FDR) after correction by the BH method was set to the threshold P.adj < 0.05, and the absolute value of the logarithmic differential expression fold change |log_2_FC|>1. The changes in differentially expressed genes at the protein level were detected by immunohistochemical data of tumor and normal tissue samples in the Human Protein Atlas [21] (HPA, http://www.proteinatlas.org). Afterward, the differentially expressed TCRGs were used to perform univariate Cox regression analysis, and the corrected threshold was set to P.adj < 0.05.

### Establishment of TRP channel clusters

For molecular subtyping, we applied consensus clustering using the “ConsensusClusterPlus” package [22] on the training set, based on the expression profiles of prognostic TCRGs. This unsupervised method repeatedly resamples both samples and features, applies clustering algorithms (here, k-means with Euclidean distance), and generates consensus matrices that reflect the stability and robustness of clustering assignments. By evaluating cumulative distribution function (CDF) plots and consensus heatmaps across a range of cluster numbers (k = 2–9), the optimal number of clusters was determined. Patients were then divided into multiple stable molecular subtypes, which represent groups with distinct gene expression patterns and potentially different clinical outcomes. Next, PCA, t-distributed stochastic neighbor embedding (tSNE) and uniform manifold approximation and projection (UMAP) were used to further evaluate the correlation of each cluster with TCRGs. Differential expression of prognostic TCRGs in different clusters was evaluated using the “limma” package. The “pheatmap” package was used to visualize the expression of TCRGs across different clusters and clinical characteristics.

### Immune infiltration and functional enrichment analysis for TRP channel clusters

Single-sample gene set enrichment analysis (ssGSEA) was used to quantify and compare the level of immune cells infiltration in samples of different subtypes, and we collected immune cell-related genes from the Gene Set Enrichment Analysis (GSEA, http://www.gsea-msigdb.org/gsea/index.jsp) website [23]. Afterward, GSVA analysis was performed on samples of different subtypes, and the “c2.cp.kegg.symbols” and “c5.go.symbols” datasets in the GSEA website were selected as the analysis gene sets to carry out [24]. KEGG and Gene Ontology [25] (GO) enrichment analysis were used to compare the enrichment of signaling pathways and biological processes among different subtypes.

### Establishment of the TRP channel-model

The “glmnet” and “survival” packages were used to perform LASSO regression analysis on prognostic TCRGs [26], and the “caret” package could randomly divide the training set samples into equal parts. The internal training and validation sets were used to select the optimal penalty parameter λ through minimum 10-fold cross-validation to construct the TRP channel-model and output the gene name and regression coefficient (coef) included in the model. The calculation formula of the prognosis model is ${\text{risk score}}=\sum\nolimits_{{i=1}}^{n} {coe{f_i}*xi}$, where x is the expression level of the gene. The risk score of each patient was calculated according to the formula, sorted from high to low, and divided into two groups (high- and low-risk) according to the median value, and the Kaplan‒Meier (KM) survival curves between two groups was drawn. Time-dependent receiver operating characteristic (ROC) curves were further used to validate the model’s efficiency and accuracy in predicting 1-, 3-, and 5-year survival. Meanwhile, we validated the predictive efficacy of the model in two external validation sets. In addition, a multivariate independent prognostic analysis was performed on the risk score and other clinical characteristics to verify the independence in predicting prognosis.

### Immune infiltration analysis for the TRP channel-model

Each sample was analysed by CIBERSORT algorithm, then a comparison was made between high- and low-risk groups in terms of immune cells infiltration [27]. At the same time, the correlation between risk scores and the expression of model genes, the infiltration of immune cells was evaluated. Furthermore, Estimate-, Immune- and Stromal scores of glioma in the training set were calculated with the ESTIMATE algorithm of the “estimate” package [28]. A higher ImmuneScore or StromalScore indicates a greater proportion of immune or stromal cells in the tumor microenvironment, and ESTIMATEScore is the sum of the two.

### Drug sensitivity analysis

To identify potentially suitable drugs for patients in different risk groups, we used the “oncoPredict” package [29] to predict patient sensitivity to drugs. Gene expression profiles of cancer cell lines (GDSC2_Expr) and corresponding drug response data (GDSC2_Res) from the Genomics of Drug Sensitivity in Cancer [30] (GDSC2, https://www.cancerrxgene.org/) database were used to build the predictive models. The drug response data were originally log-transformed half-maximal inhibitory concentrations (IC50) and were back-transformed prior to analysis. Using the calcPhenotype function, ridge regression models were applied to predict IC50 values for patients in the training set, and the predicted drug sensitivities were subsequently compared between high- and low-risk groups.

### Single-cell sequencing analysis

Glioma-related single-cell RNA sequencing (scRNA-seq) datasets (GSE148842 [31], GSE103224 [32], and GSE131928 [33]) were retrieved from the Tumor Immune Single-cell Hub 2 [34] (TISCH2, http://tisch.comp-genomics.org/). Compared with bulk RNA sequencing, which averages signals across heterogeneous tumor tissues, scRNA-seq enables cell-level resolution of transcriptomic and epigenetic features, thereby capturing cellular heterogeneity and functional states in the tumor microenvironment [35, 36]. Leveraging this advantage, we focused on the two genes with the largest absolute coefficients in our prognostic model, TRPM6 and TRPV3, and analyzed their expression patterns across distinct cell types of glioma.

### RT-qPCR validation

To validate the expression of genes included in the model, we collected paired glioma and adjacent normal tissues from six patients. All enrolled patients were pathologically diagnosed with glioma (WHO Grade 2–4) and provided written informed consent. The study was approved by the institutional ethics committee. Total RNA was extracted using the RNApure Tissue Kit (ComWin Biotech, China; Catalog No. CW0560S) and reverse-transcribed into cDNA using PrimeScript™ RT Master Mix (TaKaRa, Japan; Catalog No. RR036A). Quantitative PCR was performed using the TB Green^®^ Premix Ex Taq™ II Kit (TaKaRa, Japan; Catalog No. RR820A) on a real-time PCR instrument. GAPDH was used as the internal control. Each reaction was performed in triplicate (technical replicates). Relative expression levels were calculated using the 2^(-ΔΔCt) method. Primer sequences are listed in Supplementary Table 2.

### Statistical analysis

Statistical analysis was performed using R 4.5.0 or GraphPad Prism 10.4.2. Using t or Wilcoxon test, we compared the differences between the two groups. One-way analysis of variance (ANOVA) was used to compare multiple groups. Kaplan‒Meier method (with a log-rank test or univariate Cox regression analysis) was used to survival analysis. The correlation of two variables was analyzed using the Pearson correlation test. When the P value is less than 0.05, 0.01, 0.001, and 0.0001, it is judged to be statistically significant, which are represented by “*”, “**”, “***”, and “****”, respectively, and “ns” indicates no significance.

## Results

### Identification of prognostic TCRGs

**Fig. 2:**
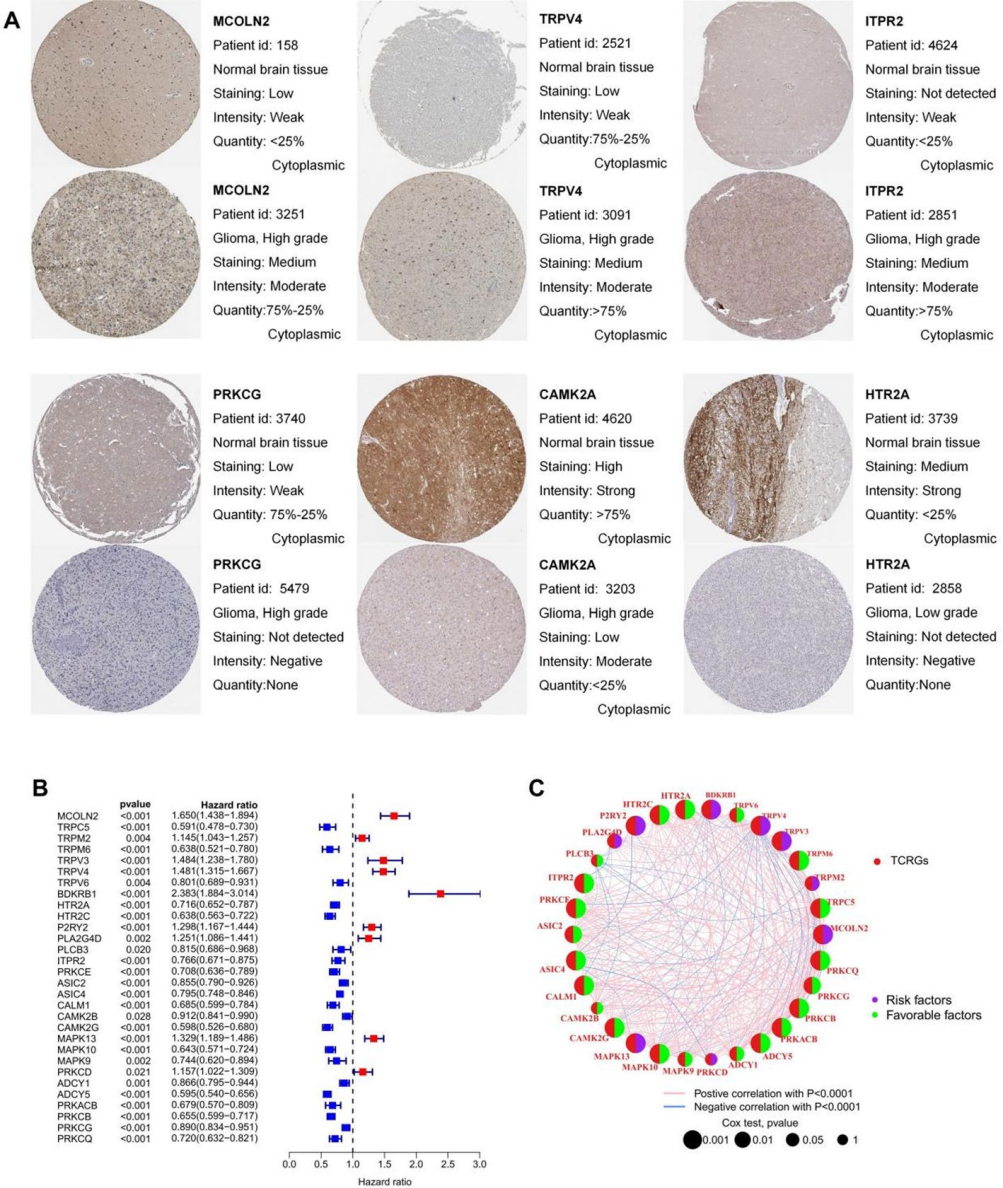
Prognostic TCRGs screening. **A** Differentially expressed TCRGs in HPA database. **B** Univariate cox regression analysis of differentially expressed TCRGs for OS. **C** A network diagram for prognostic TCRGs

Thirty-seven differentially expressed TCRG genes were obtained, with five up-regulated and 32 down-regulated (Table 1). MCOLN2, TRPV4, and ITPR2 were significantly highly expressed in glioma tissues, and the three most significantly down-regulated genes (PRKCG, CAMK2A, and HTR2A) had lower expression in tumor tissues (Fig. 2A). We found the expression of thirty genes correlated with patient survival prognosis (Fig. 2B), and a network diagram was used to visualize the intricate relationship between genes and prognostic value (Fig. 2C).

**Table 1:** Differentially expressed TCRGs

Gene id	Con-mean	Treat-mean	Log_2_FC	*P*-value	FDR
PRKCG	211.0424	16.56573438	-3.67125881	0.000198295	0.002741471
CAMK2A	738.18878	83.60561555	-3.142318057	0.000455713	0.003255093
HTR2A	39.4164	5.511879601	-2.838179738	0.000546004	0.003412522
ASIC2	15.52828	2.407887732	-2.689058005	0.000221997	0.002741471
PRKCB	153.23178	24.58510884	-2.639858893	0.000304843	0.002741471
CAMK2B	200.64352	32.29045892	-2.635454714	0.000263715	0.002741471
TRPC5	2.64922	0.428565763	-2.627979148	0.000706489	0.003723209
TRPV6	3.47302	0.599013837	-2.535529484	0.000275235	0.002741471
ADCY1	69.97276	14.27612468	-2.293188992	0.00095633	0.004157956
PLA2G4D	0.23962	0.049687589	-2.269790879	0.001381177	0.005312217
ITPR1	17.62692	3.808969044	-2.210307942	0.001553858	0.005591606
HTR2C	9.0202	2.164219971	-2.05931228	0.000286938	0.002741471
TRPM6	3.57034	0.861622682	-2.050933333	0.007404011	0.018510028
PRKCE	60.31824	14.69733381	-2.037039869	0.000430043	0.003255093
CALM3	1086.28142	284.1873961	-1.93448343	0.000297174	0.002741471
ADCY5	61.95748	17.71862981	-1.806011422	0.000147051	0.002741471
BDKRB1	0.48794	0.140958203	-1.791436323	0.00048948	0.003263197
PLA2G4E	0.04852	0.015015264	-1.692149718	0.002205242	0.007350806
CALM1	1292.71966	430.220904	-1.587259915	0.000261465	0.002741471
MAPK13	9.64888	3.267023538	-1.562384641	0.001833101	0.006321038
MAPK9	77.78872	26.38899715	-1.559624442	0.000210746	0.002741471
PRKCQ	19.35522	7.2509602	-1.416478746	0.008883946	0.02066034
TRPV3	3.82662	1.46571398	-1.384467039	0.002288397	0.007381925
PRKACB	250.45698	98.88096919	-1.34079803	0.000183301	0.002741471
PRKCD	50.77336	20.65019743	-1.297916161	0.000791122	0.003771537
TRPM2	24.27016	10.19642325	-1.251120454	0.003190356	0.009969863
KNG1	0.09846	0.04466291	-1.140460483	0.024108628	0.047790936
TRPA1	0.1876	0.087833381	-1.094818586	0.011074598	0.024610218
MAPK10	19.64456	9.233521541	-1.089176972	0.004918255	0.01329258
CAMK2G	128.71258	60.80652611	-1.08185499	0.003779987	0.011454506
P2RY2	0.63052	0.299850214	-1.072300131	0.004687655	0.013021263
MAPK11	80.19096	39.97856334	-1.004212981	0.004255797	0.012517049
ITPR2	9.30202	23.66173096	1.34693967	0.004560442	0.013021263
PLCB3	9.9037	27.67899715	1.482752154	0.000328977	0.002741471
TRPV4	0.23142	1.331616262	2.524592938	0.00070741	0.003723209
MCOLN2	0.14968	1.072156348	2.840561935	0.008770055	0.02066034
ASIC4	8.97516	68.61931755	2.934605214	0.012119017	0.025785142

### Establishment of TRP channel clusters

**Fig. 3:**
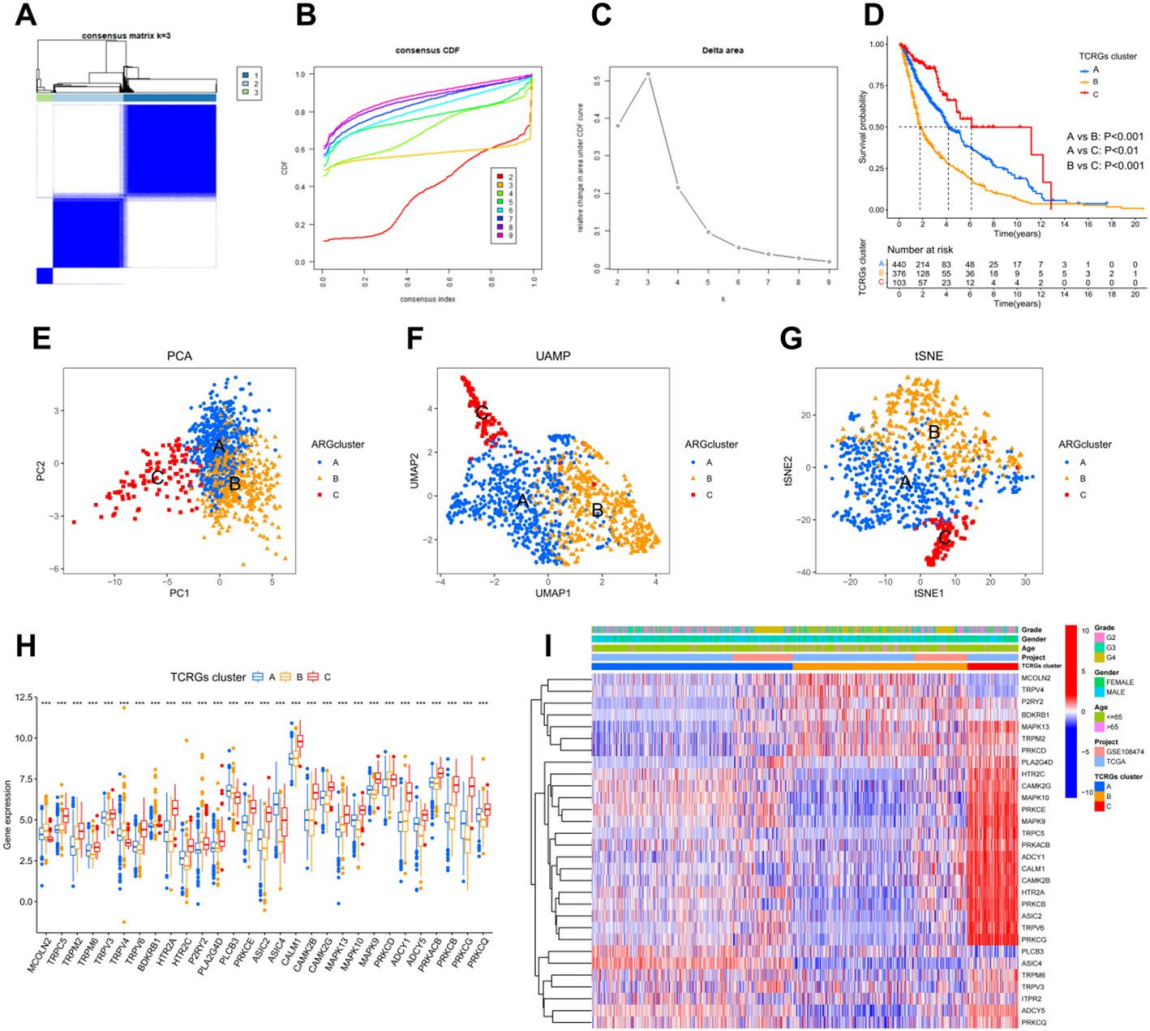
Establishment of TRP channel clusters. **A** Consensus clustering matrix when k = 3. **B**, **C** Representative cumulative distribution function (CDF) curves (**B**), CDF delta area curves (**C**). **D** Survival analysis between different clusters. **E**–**G** PCA (**E**), UMAP (**F**) and tSNE (**G**) visualizes the distribution of three clusters. **H** Expression of prognostic TCRGs in different clusters. **I** Expression of prognostic TCRGs in different clinical characteristics

Using the consensus clustering algorithm of the “ConsensusClusterPlus” package, we identified three subtypes associated with prognostic TCRGs (Fig. 3A and C). Survival analysis showed that Cluster B had the worst prognosis, while Cluster C had longer survival (Fig. 3D). PCA, UMAP, and tSNE analyses further validated the clustering of patients in the three subtypes, confirming its reliability and stability (Fig. 3E and G). A total of 29 prognostic TCRGs were differentially expressed in different clusters, and these genes may be effective biomarkers for cluster analysis (Fig. 3H). We also found that Cluster C mainly came from younger WHO Grade 2–3 patients, which may account for its better prognosis (Fig. 3I).

### Immune infiltration and functional enrichment analysis for TRP channel clusters

**Fig. 4:**
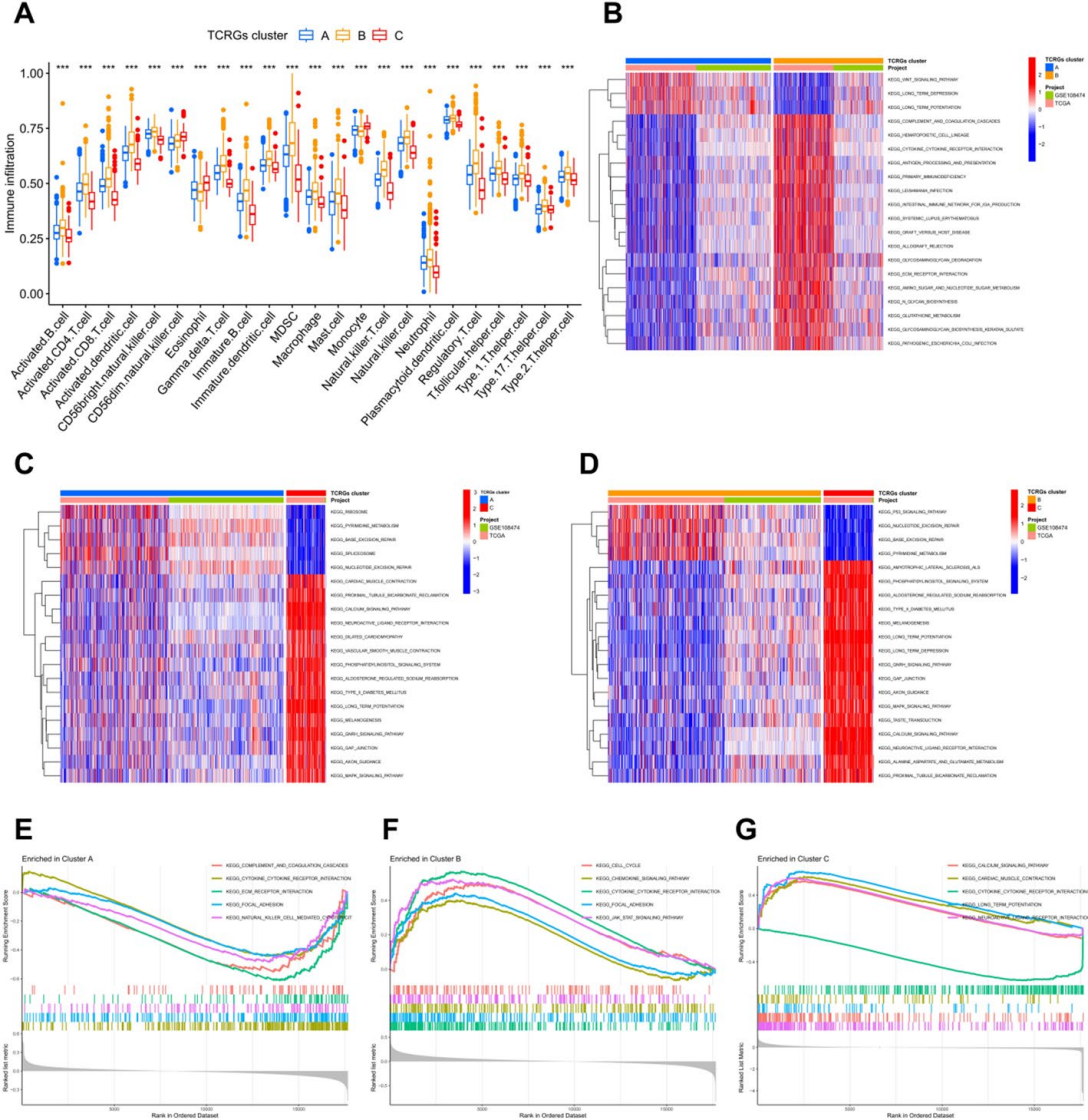
Immune infiltration and enrichment analysis for TRP channel clusters. **A** Differential expression of 22 immune cells in different clusters. **B**–**D** Differences in the KEGG pathway enrichment analysis among different clusters. **E**–**G** GSEA enrichment analysis results of different clusters

ssGSEA showed that most immune cells were significantly enriched in Cluster B, such as activated B cells, CD8^+^ T cells, CD4^+^ T cells, macrophages, mast cells, natural killer T cells, neutrophils and type 17 T helper cells. In Cluster C, with a better prognosis, the infiltration level of most immune cells was lower (Fig. 4A). Although the prognosis of Cluster B patients was worse, we found that their higher level of immune cells infiltration indicates that they were in an immunosuppressive tumor microenvironment, and more aggressive immunotherapy is appropriate for these patients. Next, we performed KEGG pathway enrichment analysis between each subtype. Taking Cluster B and Cluster C with the greatest difference in survival prognosis as an example, we found that the following pathways were significantly activated in Cluster C: phosphatidylinositol signaling system, gap junction, GNRH signaling pathway, axon guidance, MAPK signaling pathway, neuroactive ligand receptor interaction, calcium signaling pathway, etc. The P53 signaling pathway, base excision repair, nucleotide excision repair, pyrimidine metabolism, etc. were significantly enriched in Cluster C (Fig. 4B and D). GSEA analysis found some tumor progression related pathways were significantly enriched in Cluster B: cell cycle, cytokine‒cytokine receptor interaction, focal adhesion, JAK-STAT signaling pathway and chemokine signaling pathway (Fig. 4E and G).

### Establishment of the TRP channel-model

**Fig. 5:**
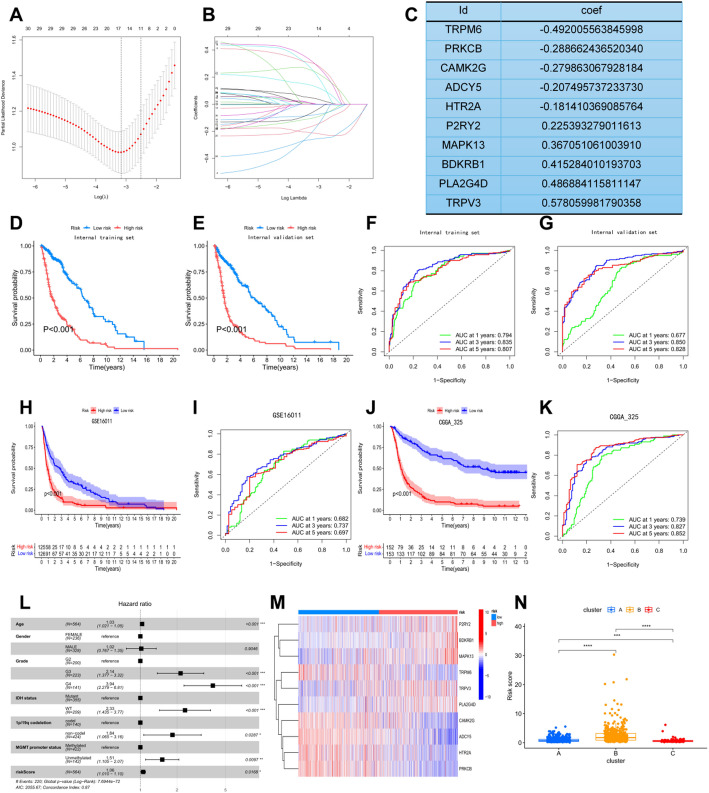
Construction and validation of the prognostic model. **A**, **B** LASSO Cox regression model construction. **C** The coef of each risk factor included in the model. **D**, **E** The K-M curves for survival in internal training and validation sets. **F**, **G** The ROC curves of the prognostic model in predicting 1-, 3-, and 5-year OS in internal training and validation sets. **H**, **J** The K-M curves for survival in GSE16011 and CGGA_325 cohorts. **I**, **K** The ROC curves of the prognostic model in predicting 1-, 3-, and 5-year OS in GSE16011 and CGGA_325 cohorts. **L** Multivariate independent prognostic analysis for the risk score. **M** Expression of genes included in the model between the high- and low- risk groups. **N** Expression of the risk score in different clusters

A total of 10 genes were included in the model (Fig. 5A and C). In the internal training and validation set, we found that the OS of patients in the high-risk group was significantly lower than that in the low-risk group (*P* < 0.001; Fig. 5D, E). The ROC curve demonstrated that the model had good predictive performance for the OS of patients at 1, 3, and 5 years (Fig. 5F, G). The same results were obtained in two validation sets (*P* < 0.001; Fig. 5H–K). Multivariate independent prognostic analysis found that risk score, age, WHO classification, IDH status, 1p/19q codeletion, and MGMT promoter status were all indicators of survival in glioma patients (Fig. 5L). According to the model, TCRGs expression differed between two groups (high- and low-risk) in Fig. 5M. Meanwhile, we found that Cluster B had a higher risk score, while Cluster C had the lowest risk score, which was consistent with the survival of patients with different subtypes (Fig. 5N).

### Immune infiltration analysis for the TRP channel-model

**Fig. 6:**
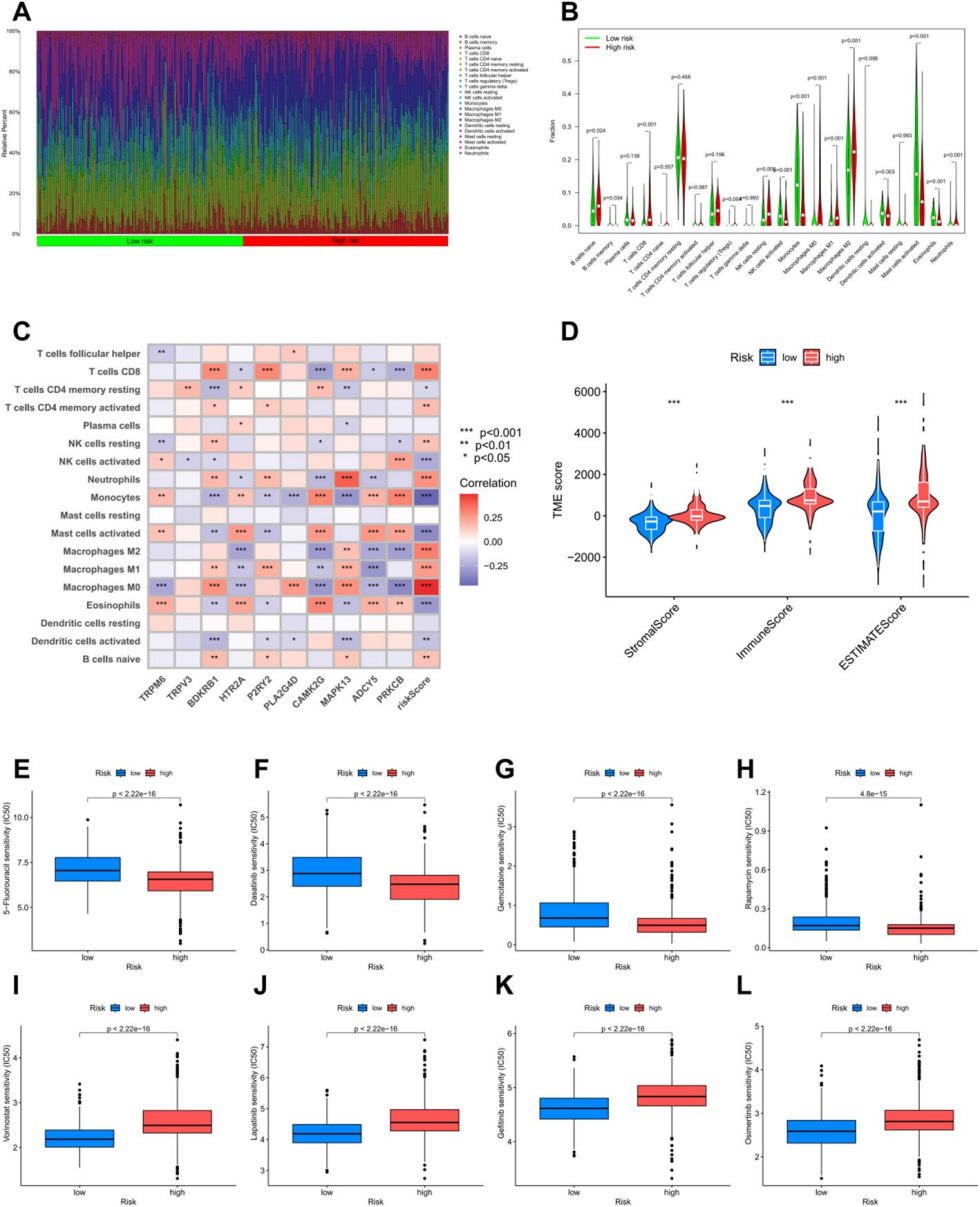
Immune infiltration and drug sensitivity analyses for the TRP channel-model. **A**, **B** Expression of 22 immune cells in the high- and low- risk groups. **C** Correlation between the prognostic model and the degree of immune cell infiltration. **D** ESTIMATE algorithm for the prognostic model. In high-risk patients, the four most sensitive drugs were (**E**) 5-Fluorouracil, **F** Dasatinib, **G** Gemcitabine, and (**H**) Rapamycin. In low-risk patients, the four most sensitive drugs were (**I**) Vorinostat, **J** Lapatinib, **K** Gefitinib, and (**L**) Osimertinib

By analyzing the immune microenvironment, we found that most immune cells had different infiltration conditions between two groups, such as naive B cells, resting NK cells, CD8^+^ T cells, activated NK cells, monocytes, M0, M1 and M2 macrophages, activated mast cells, eosinophils, and neutrophils (Fig. 6A, B). It was further found that TCRGs expression and the risk score showed potential associations with immune cell infiltration, which may provide insights into the involvement of these genes in microenvironmental alterations (Fig. 6C). The ESTIMATE algorithm verified the above findings, and relevant scores were significantly higher in patients of the high-risk group (Fig. 6D).

### Drug sensitivity analysis

Based on predicted IC50 values using the “oncoPredict” package, we identified 141 drugs exhibiting significant differences in sensitivity between high- and low-risk patient groups, among which 36 drugs have been approved by the FDA (Supplementary Table 3). In high-risk patients, the four most sensitive drugs were 5-Fluorouracil, Dasatinib, Gemcitabine, and Rapamycin (Fig. 6E–H), whereas in low-risk patients, Vorinostat, Lapatinib, Gefitinib, and Osimertinib showed the greatest predicted sensitivity (Fig. 6I–L). These findings suggest that the risk model can guide personalized therapy by identifying candidate drugs that may be more effective for patients in specific risk groups, potentially improving treatment outcomes.

### Single-cell sequencing analysis

**Fig. 7:**
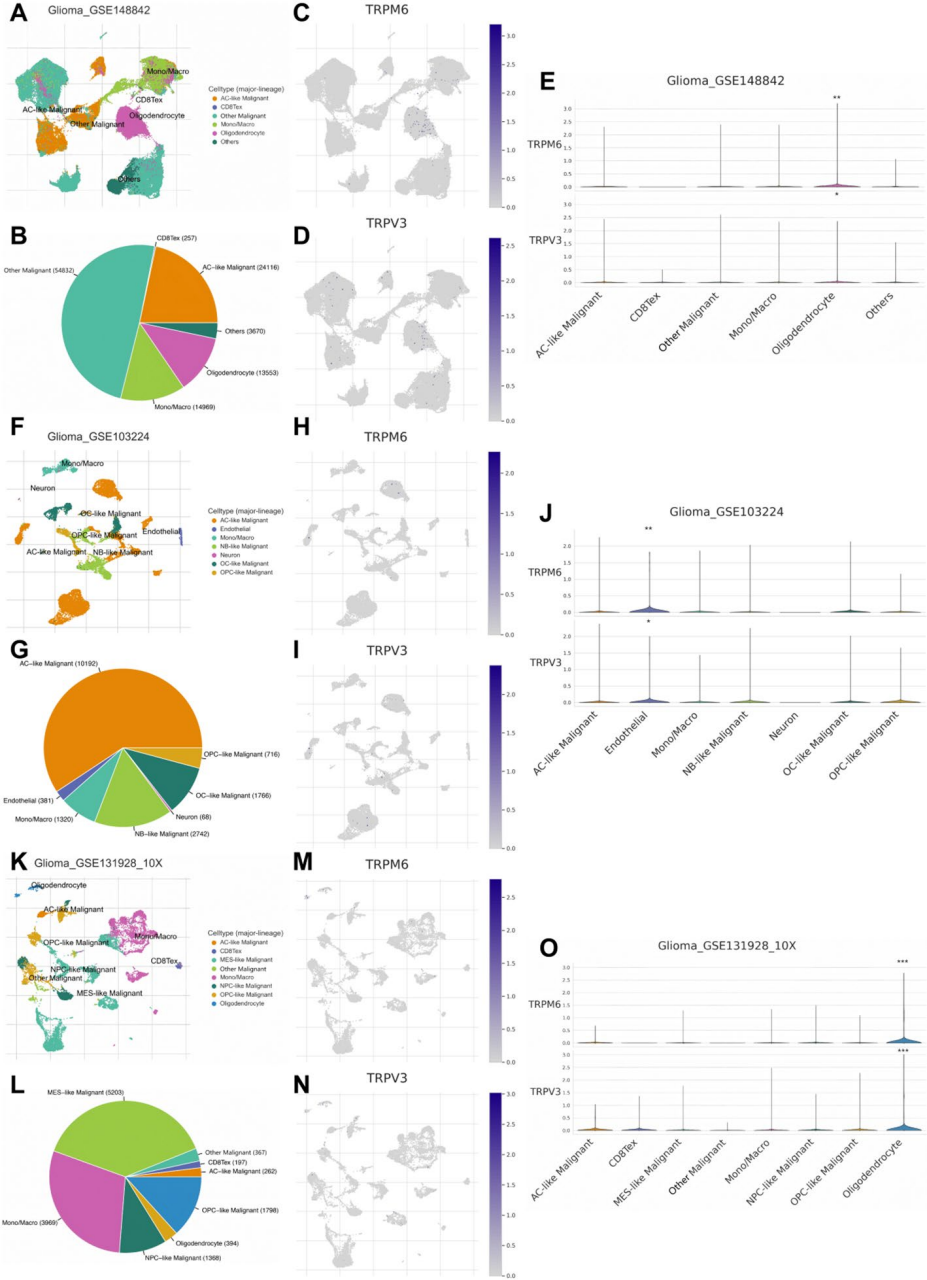
scRNA-seq analysis of TRP channel-related genes (TRPM6 and TRPV3) across multiple datasets from the TISCH2 database. **A**, **F**, **K** UMAP plots showing annotated cell types in three datasets. **B**, **G**, **L** The total numbers of cells in each dataset. **C**, **H**, **M** The expression localizations of TRPM6 in different datasets. **D**, **I**, **N** The expression localizations of TRPV3 in different datasets. **E**, **J**, **O** The expression patterns of TRPM6 and TRPV3 in different datasets

In the TISCH2 database, scRNA-seq datasets were processed and analyzed using the “MAESTRO” package [37]. Briefly, quality control was performed to remove low-quality cells and potential doublets. Dimensionality reduction and clustering were conducted to identify distinct cellular populations, followed by cell-type annotation based on known marker genes (Fig. 7A, F, K). Across the three datasets, a total of 111,397 (Fig. 7B), 17,185 (Fig. 7G), and 13,553 (Fig. 7L) cells were identified, with malignant cells representing the largest proportion in all datasets. In the GSE148842 and GSE131928 datasets, TRPM6 and TRPV3 were more highly expressed in oligodendrocytes compared to other cells (Fig. 7E, O). In the GSE103224 dataset, their expression was higher in endothelial cells (Fig. 7J). These observations are consistent with our previous analysis showing that most TCRGs are expressed at lower levels in tumor tissues than in normal tissues (Table 1). Furthermore, in the GSE131928 dataset, TRPV3 was expressed in exhausted CD8^+^ T cells, whereas TRPM6 was nearly absent. Exhausted CD8^+^ T cells are hallmark immunosuppressive cells in the tumor microenvironment and are generally associated with poor patient prognosis. In our prognostic model, TRPV3 was identified as a significant risk factor, while TRPM6 acted as a protective factor, highlighting the model’s effectiveness in predicting patient outcomes and reflecting immune infiltration.

### RT-qPCR validation

**Fig. 8:**
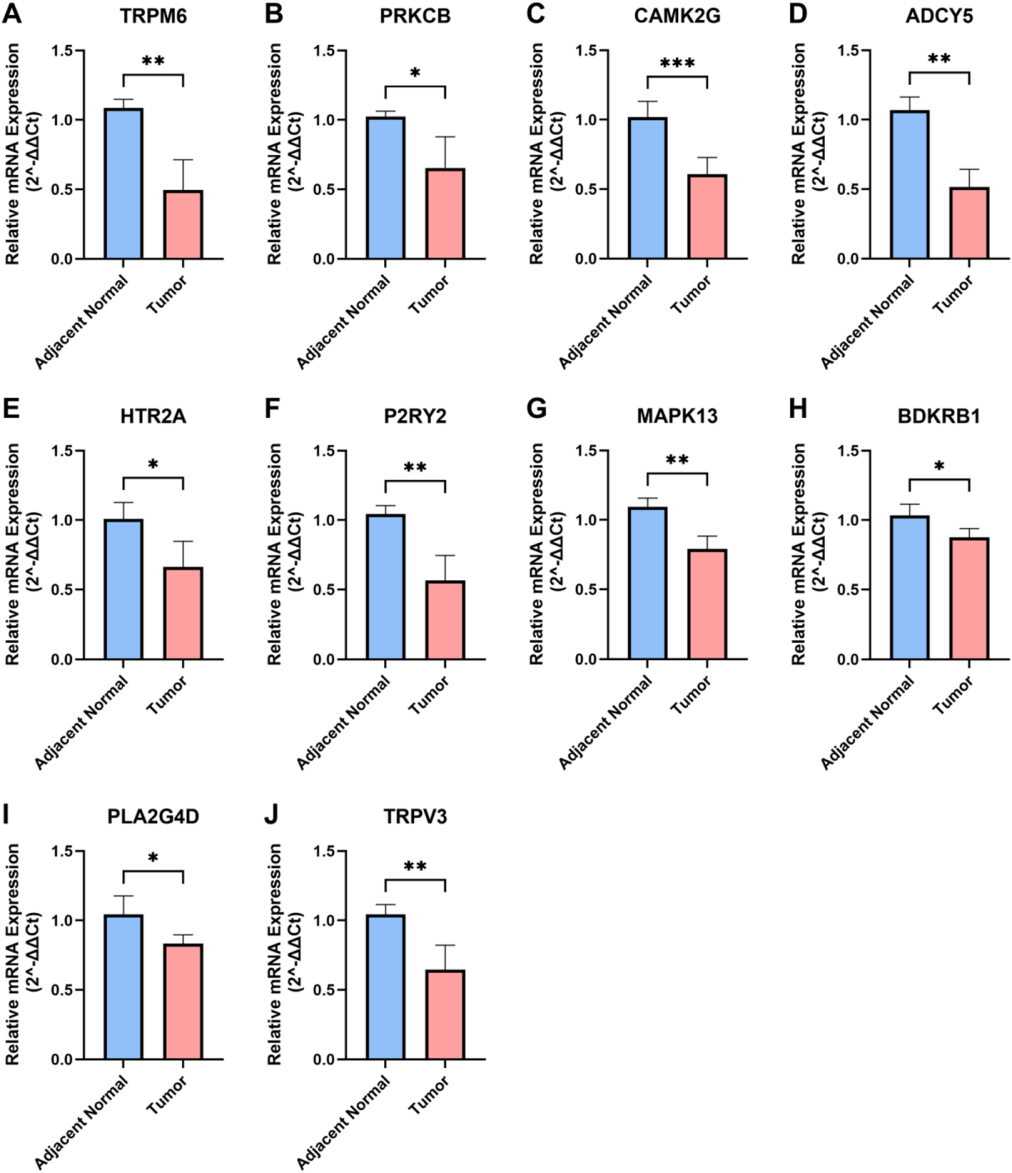
Validation of model genes in glioma and adjacent normal tissues by RT-qPCR. **A** TRPM6, **B** PRKCB, **C** CAMK2G, **D** ADCY5, **E** HTR2A, **F** P2RY2, **G** MAPK13, **H** BDKRB1, **I** PLA2G4D, and **J** TRPV3. Data are presented as mean ± SD

To validate our prognostic model, we performed RT-qPCR on paired glioma and adjacent normal tissues from six patients. The results showed that all 10 genes included in the model were expressed at lower mRNA levels in glioma tissues compared with the corresponding peritumoral normal tissues (Fig. 8A–J).

## Discussion

TRP channels are cation channels on the cell membrane surface that allow cations such as Ca^2+^, Mg^2+^, Na^+^, and K^+^ to pass through. In the tumor microenvironment, an imbalance in the expression level of TCRGs can change the adaptability of cells to the surrounding environment and regulate the occurrence and development of tumors. Research shows that blocking or interfering with TRP channels is expected to become a new target for tumor molecular therapy. A pan-cancer analysis found that higher WHO grade and stage were substantially correlated with unbalanced TCRG expression, and shorter survival, increased tumor mutational burden, and activation of tumor-related pathways in patients [38]. Previous studies have found that TRPML1 blocks the autophagic flux of human glioblastoma cells to lysosomes by inducing autophagy inhibition, resulting in the accumulation of damaged mitochondria, which in turn damages DNA in cancer cells and inhibits tumor growth [39]. Another study reported that elevated expression levels of TRPML2 were associated with resistance to temozolomide therapy in GBM patients and were associated with poorer OS. Interfering with TRPML2 expression was expected to be a new therapeutic target [40]. However, there is no literature systematically evaluating the potential prognostic value and mechanism of TCRGs in glioma, so our study has certain significance.

In this study, 37 differentially expressed TCRGs in glioma tissues were first identified by differential analysis, and 30 genes were further screened by univariate Cox regression analysis to associate with patient survival and prognosis. Due to the complex molecular heterogeneity of glioma, the identification of different molecular subtypes can help develop better treatment options [41]. To this end, based on 30 prognostic TCRGs, we used the consensus clustering algorithm of the “ConsensusClusterPlus” package, selected the optimal number of clusters (k = 3), and identified three subtypes, which were confirmed reliability by PCA, UMAP, and tSNE. We found that Cluster B patients had the worst prognosis, and patients in Cluster C had the longest survival. Further biological and immune-related analyses found that Cluster B had a higher degree of immune infiltration and a higher level of immunosuppression. At the same time, multiple signaling pathways related to cancer progression were significantly enriched, which may partly explain the poor prognosis of this subtype. Our subtype identification method provides reference for the molecular typing of glioma.

Although the WHO classification method has been the gold standard for prognostic grading of glioma for many years, the identification of more tumor biomarkers based on bioinformatics methods and a large amount of gene sequencing data has become a new technological advancement and contributes to precise medical realization. The prognostic value of a single biomarker is very limited, and integrating multiple biomarkers into one model can improve the prediction efficiency and accuracy [42]. For example, a study constructed a prognostic model for glioma patients based on three necroptosis-related genes, and high-risk patients had higher immune infiltration rates and immune checkpoint genes expression than low-risk patients, which were positively associated with poor prognosis [43]. Another study constructed a prognostic model in patients with LGG based on cellular senescence-related genes and found that for patients, cellular senescence-related scores could be used as an independent predictor, and patients with high scores had a worse prognosis. It is also well validated in other cohorts [44]. We constructed a 10-gene prognostic model, and patients were divided into two groups. The findings indicated that the OS of patients in the high-risk was significantly lower, and two external validation sets (CGGA_325 and GSE16011) corroborated this finding favorably. Then we performed an immune microenvironment analysis. The results showed that the infiltration of immune cells was higher in patients of the high-risk group. The coexistence of immune-suppressive and immune-activating cells suggested that in the high-risk group, there are complex immune microenvironment disturbances in patients, and effective immune intervention is expected to improve the survival of such patients.

In addition to prognostic stratification, our risk model also provided guidance for personalized therapy. Drug sensitivity analysis identified a series of drugs that were more effective in either the high- or low-risk groups. For example, Dasatinib, a small-molecule tyrosine kinase inhibitor, has been shown in vitro to effectively suppress the malignant phenotype of glioma stem cells when combined with a WEE1 inhibitor, and it exhibited higher predicted sensitivity in the high-risk group [45]. In the low-risk group, Vorinostat demonstrated greater sensitivity, and studies have indicated that it can effectively prevent postoperative recurrence of glioma [46].

Our model included a total of 10 genes, all of which were downregulated in glioma tissues compared with normal brain tissues. TRPM6 encodes a channel kinase that regulates Mg^2+^ homeostasis through intestinal absorption and renal reabsorption. Although direct evidence in glioma is limited, studies in other cancers (e.g., neuroblastoma) suggest that TRPM6 expression can be upregulated by oncogenes such as N-Myc, thereby influencing ionic balance and cell proliferation [47].

PRKCB, a member of the serine- and threonine-specific protein kinase C family, is crucial for B-cell receptor signaling and development, and its dysregulation has been associated with chronic lymphocytic leukemia [48, 49]. It also promotes hepatocellular carcinoma progression through interaction with RACK1 [50] and contributes to tumor immune escape by impairing dendritic cell differentiation via STAT3 signaling [51].

CAMK2G is a Ca²⁺/calmodulin-dependent kinase that regulates multiple oncogenic processes through phosphorylation-dependent mechanisms. It has been shown to stabilize c-Myc via S62 phosphorylation, promote STAT3 and ERK signaling, and support tumor growth in multiple myeloma and colitis-associated colorectal cancer, as well as contribute to cisplatin resistance through redox adaptation [52]. Inversely, CAMK2G deficiency can accelerate mTORC1-driven hepatocarcinogenesis, highlighting its context-dependent dual role similar to NF-κB in the liver [53]. In neuroblastoma, high CAMK2G expression correlates with favorable prognosis and neuronal differentiation, whereas its loss promotes proliferation and invasion [54].

ADCY5 is a cAMP-producing enzyme that regulates cellular proliferation, migration, and EMT through inhibition of AKT, PKG, and Wnt signaling. Its expression is frequently downregulated in glioblastoma and other cancers, and higher ADCY5 levels are associated with better prognosis. Functional studies suggest that ADCY5 acts as a tumor suppressor in GBM by restricting cell growth, invasion, and stemness, potentially modulated by epigenetic methylation [55].

HTR2A is a gene encoding the serotonin receptor 5-HT2A, a G protein-coupled receptor involved in various central nervous system functions. In glioma, HTR2A expression is associated with tumor heterogeneity and immune cell infiltration, potentially influencing patient prognosis and the effectiveness of immunotherapies. Additionally, HTR2A has been implicated in neuroactive ligand–receptor interactions and calcium signaling pathways, which may play roles in glioma pathogenesis [56].

P2RY2 encodes a purinergic G protein-coupled receptor that responds to extracellular ATP/UTP and regulates calcium-dependent signaling pathways. Aberrant activation of P2RY2 has been implicated in several cancers by promoting proliferation, invasion, angiogenesis, and immune evasion [57]. In glioma, elevated P2RY2 expression enhances tumor invasiveness and is associated with poor prognosis, suggesting its potential as a prognostic biomarker and therapeutic target [58].

MAPK13 is a subtype of the p38 mitogen-activated protein kinase family, which is highly conserved and involved in the regulation of inflammatory responses, cell proliferation [59, 60]. Studies have confirmed that MAPK13 promotes the over-proliferation of epidermal cells and tumorigenesis by inhibiting the expression of proinflammatory cytokines and chemokines in the development of squamous cell carcinoma [61].

BDKRB1 encodes the inducible bradykinin B1 receptor, a G-protein–coupled receptor that mediates bradykinin-driven inflammatory responses, vascular permeability and angiogenic signaling. In glioma, bradykinin induces IL-8 expression through the bradykinin B1 receptor and promotes GBM migration [62]. The expression level of BDKRB1 gradually increases with higher WHO grades of glioma, suggesting that it may serve as a risk factor for patients [63].

PLA2G4D encodes cytosolic phospholipase A2 group IVD, an sn-2 glycerophospholipid hydrolase that liberates free fatty acids (including arachidonic acid) and lysophospholipids from membrane phospholipids. Products of PLA2 activity feed eicosanoid biosynthesis and modulate inflammation, proliferation and cell migration—pathways widely implicated in cancer biology [64].

TRPV3 mainly exists in sensory nerve cells and skin keratinocytes in the human body and senses temperature stimuli by forming a complex with TRPV1 [65]. Studies have shown that in renal clear cell carcinoma and non-small cell lung cancer, the high expression of TRPV3 is significantly correlated with increased tumor grade and poor prognosis [66, 67]. In renal clear cell carcinoma, TRPV3 can induce the expression of immune checkpoints such as LAG3, CTLA4, PDCD1, and TIGIT, leading to the accumulation of immunosuppressive T cells in the tumor microenvironment. In our single-cell sequencing data analysis, we also observed that the risk gene TRPV3 was expressed in exhausted CD8^+^ T cells, whereas the protective factor TRPM6 was not, further supporting that TRPV3 may be involved in the induction and accumulation of exhausted CD8^+^ T cells within the tumor microenvironment.

Our study firstly explored the prognostic value of TRP channel-related genes in glioma, which has certain innovation and research value. However, this is a bioinformatics study based on an open databases, so a larger multicenter studies are needed to confirm the validity of the constructed prognostic model, and sufficient validation and mechanistic exploration are needed, which will be included in the follow-up experimental design. We hope that through the existing research results, more researchers will be attracted to participate in research on the correlation between the TRP channel and the mechanism of the occurrence and development of glioma and provide a more theoretical basis for the development of therapeutic targets for this type of tumor.

## Limitations and future directions

Despite providing a novel prognostic framework, our study relies on publicly available datasets with potential selection bias, and lacks extensive in vitro and in vivo functional validation of key TRP channel genes. Future endeavors should include large-scale, multicenter cohorts to confirm these findings, along with mechanistic investigations into how TRP channel aberrations influence glioma immune microenvironment and therapeutic resistance. Additionally, integrating multi-omic analyses (e.g., genomics, proteomics, and metabolomics) and exploring targeted combination therapies could further refine personalized treatment strategies, ultimately improving clinical outcomes and guiding the development of more effective interventions against glioma.

## Conclusion

Based on the expression of TRP channels-related genes, we conducted the new subtype classification and a prognostic model for glioma, which can effectively predict the survival prognosis and tumor microenvironment state of patients, and is expected to provide theoretical basis for the development of new targets.

## Supplementary Information

Supplementary material 1.

Supplementary material 2.

Supplementary material 3.

Supplementary material 4.

## Data Availability

The datasets analyzed in this study were obtained from publicly accessible databases, including TCGA (https://portal.gdc.cancer.gov/), CGGA (http://www.cgga.org.cn/), and multiple GEO datasets. Specifically, bulk transcriptomic datasets of glioma were retrieved from GEO under accession numbers GSE108474 and GSE16011, while single-cell RNA-sequencing datasets were incorporated from GSE148842, GSE103224, and GSE131928. Additional data resources were obtained from HPA (http://www.proteinatlas.orgdatabases) and TISCH2 (http://tisch.comp-genomics.org/) databases.

## Abbreviations

TRP: Transient receptor potential
TCRGs: TRP channel-related genes
LGG: Low grade glioma
GBM: Glioblastoma
OS: Overall survival
TCGA: The Cancer Genome Atlas
GEO: Gene Expression Omnibus
CGGA: Chinese Glioma Genome Atlas
MsigDB: Molecular Signatures Database
KEGG: Kyoto Encyclopedia of Genes and Genomes
GO: Gene Ontology
FDR: False discovery rate
HPA: Human Protein Atlas
CDF: Cumulative distribution function
PCA: Principal component analysis
tSNE: T-distributed stochastic neighbor embedding
UMAP: Uniform manifold approximation and projection
ssGSEA: Single-sample gene set enrichment analysis
GSEA: Gene Set Enrichment Analysis
KM: Kaplan‒Meier
ROC: Receiver operating characteristic
GDSC2: Genomics of Drug Sensitivity in Cancer
IC50: Half-maximal inhibitory concentration
scRNA-seq: Single-cell RNA sequencing
TISCH2: Tumor Immune Single-cell Hub 2
ANOVA: One-way analysis of variance
