# Supplementary figures and images for: A novel TRP channel-related prognostic model of glioma based on transcriptomics and single cell sequencing analysis
Source: Discov Oncol. 2025 Dec 2;17:29. doi: 10.1007/s12672-025-04220-5 (PMC12775248; doi:10.1007/s12672-025-04220-5)

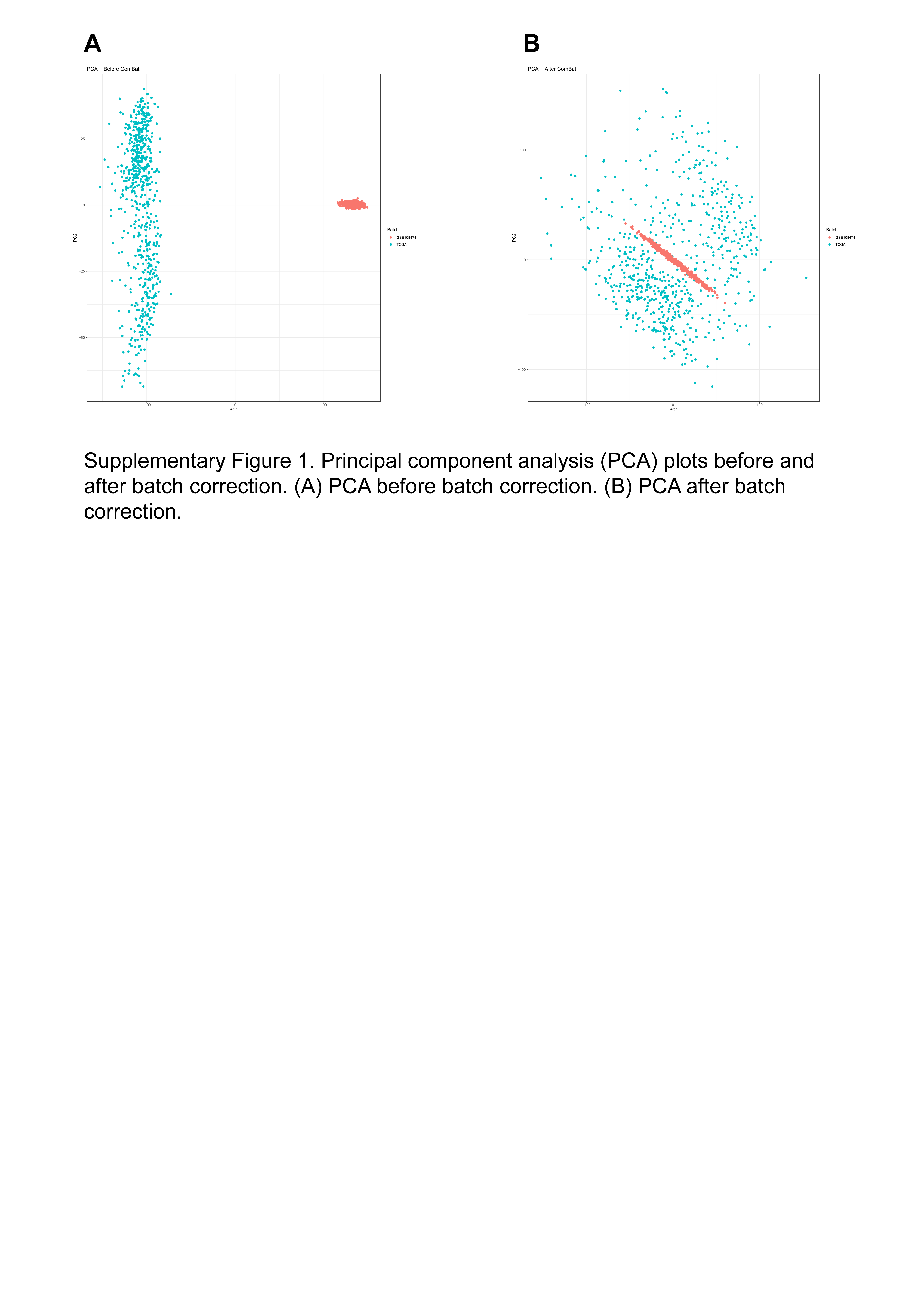

Supplement: Supplementary file 1 — Supplementary material 1. [file 12672_2025_4220_MOESM1_ESM.png]
